# Distinct Clinical Phenotypes of Infection and Mechanical Dysfunction in Ventriculoperitoneal Shunt Complications

**DOI:** 10.7759/cureus.103824

**Published:** 2026-02-18

**Authors:** Hasan Sener, Yunus Emre Durmuş, Cengiz Çokluk

**Affiliations:** 1 Department of Neurological Surgery, Ankara Gazi Mustafa Kemal Occupational and Environmental Diseases Hospital, Ministry of Health, Ankara, TUR; 2 Department of Neurosurgery, Faculty of Medicine, Ondokuz Mayıs University, Samsun, TUR

**Keywords:** abdominal ultrasonography, hydrocephalus, mechanical shunt dysfunction, papilledema, shunt infection, ventriculoperitoneal shunt

## Abstract

Introduction

Ventriculoperitoneal shunt complications, particularly infection and mechanical dysfunction, represent major causes of morbidity and revision surgery in patients with hydrocephalus.

Objective

This study aimed to evaluate our single-center experience with ventriculoperitoneal shunt revisions and to compare shunt infection and mechanical shunt dysfunction in order to identify institution-specific clinical, laboratory, and cerebrospinal fluid (CSF) characteristics that may facilitate differential diagnosis.

Results

Among the patients, 140 (35.9%) were diagnosed with ventriculoperitoneal shunt infection, while 250 (64.1%) had mechanical shunt dysfunction. Fever, elevated C-reactive protein (CRP) and white blood cell (WBC) levels, reduced CSF glucose, and increased CSF protein levels were significantly more frequent in the infection group (p<0.001). Shunt infections predominantly occurred in the early postoperative period, whereas mechanical shunt dysfunction was more commonly observed during the late postoperative period. Coagulase-negative staphylococci were the most frequently isolated pathogens in CSF cultures.

Conclusion

Ventriculoperitoneal shunt infection and mechanical shunt dysfunction demonstrate clearly distinct clinical and biochemical profiles. Early assessment of these parameters may facilitate more accurate differentiation and support rational clinical decision-making.

## Introduction

Hydrocephalus is a heterogeneous clinical condition characterized by ventricular dilatation resulting from an imbalance between cerebrospinal fluid (CSF) production and absorption, with etiologies ranging from congenital anomalies to acquired disorders [1,2]. Obstruction of CSF pathways, impaired venous absorption, and, less commonly, excessive CSF production constitute the main mechanisms underlying hydrocephalus development [3].

Congenital anomalies and perinatal hemorrhage are the predominant causes in congenital cases, whereas infection, subarachnoid hemorrhage, and trauma represent the most common etiological factors in acquired hydrocephalus [2,4,5].

Ventriculoperitoneal shunt placement remains one of the most frequently used surgical treatment modalities for hydrocephalus [6]. Long-term follow-up studies have demonstrated high rates of mechanical complications and infections, with reported shunt failure rates ranging from 11% to 25% within the first postoperative year [7-9]. Consequently, a considerable proportion of patients require multiple shunt revisions during their lifetime [8-11].

Shunt infection represents a serious complication, typically characterized by alterations in CSF biochemistry, positive culture results, and elevated systemic inflammatory markers. Most infections arise from skin flora microorganisms that colonize the shunt material and predominantly occur during the early postoperative period [12,13]. The higher incidence of shunt infections observed in pediatric patients has been attributed to immunological immaturity and differences in skin flora composition [12,14,15]. These infections are frequently associated with increased morbidity and may necessitate external ventricular drainage (EVD), prolonged antibiotic therapy, and complete shunt removal [12,16].

In contrast, shunt dysfunction encompasses a broad spectrum of complications that usually develop independently of infection, including mechanical obstruction, valve malfunction, distal catheter obstruction, pseudocyst formation, and disturbances in hydrodynamic flow [17]. Shunt dysfunction has been reported more frequently in patients with congenital hydrocephalus, largely due to the requirement for shunt placement at an early age [18]. Clinically, shunt dysfunction presents with ventricular enlargement, headache, vomiting, neurological deterioration, and loss of reservoir function, while laboratory findings are generally not indicative of infection.

Accurate clinical, laboratory, and radiological differentiation between shunt infection and shunt dysfunction is essential for appropriate therapeutic decision-making, avoidance of unnecessary surgical interventions, and reduction of morbidity. While shunt infection requires urgent surgical management and antimicrobial therapy, the primary therapeutic goal in shunt dysfunction is the correction of mechanical obstruction. Therefore, careful evaluation of the distinguishing features of these two conditions plays a critical role in clinical management.

## Materials and methods

Study design, patient selection, and grouping

This study was designed as a retrospective, single-center observational analysis conducted at Ondokuz Mayıs University, a tertiary neurosurgical referral center in Samsun, Turkey. Medical records of patients who underwent ventriculoperitoneal shunt surgery for the treatment of hydrocephalus at the Department of Neurosurgery, Faculty of Medicine, Ondokuz Mayıs University, between 2013 and 2022 were reviewed.

During the study period, a total of 1,206 patients underwent ventriculoperitoneal shunt placement. Among these, 390 patients who required surgical revision due to shunt-related complications were included in the study. Patients were eligible if they had a diagnosis of hydrocephalus treated with ventriculoperitoneal shunt, underwent shunt revision for either infection or mechanical dysfunction, and had available laboratory and CSF data. Patients in whom CSF diversion methods other than ventriculoperitoneal shunt were used, such as ventriculosubgaleal shunting or standalone endoscopic third ventriculostomy, were excluded.

Patients requiring surgical revision were divided into two distinct groups based on the indication for revision: ventriculoperitoneal shunt infection and mechanical shunt dysfunction. This classification was selected because it reflects the primary clinical decision-making pathway in shunt-related complications and directly influences surgical strategy and postoperative management.

Shunt infection was defined by the presence of clinical signs of infection, elevated inflammatory markers, abnormal CSF biochemistry and/or microbiological findings, and intraoperative evidence of infection leading to shunt removal or revision. Mechanical shunt dysfunction was defined as shunt failure due to obstruction, valve malfunction, or distal catheter-related problems in the absence of clinical, laboratory, or microbiological evidence of infection.

By grouping patients according to these criteria, the study aimed to compare two clinically distinct phenotypes of shunt-related complications and to identify parameters that may facilitate accurate differential diagnosis in routine clinical practice.

Ethics committee approval was obtained from the Faculty of Medicine of Ondokuz Mayıs University (approval number: E-15374210-811.99-2300038271) prior to study initiation, and all procedures were conducted in accordance with the principles of the Declaration of Helsinki.

Statistical analysis

Statistical analyses were performed using IBM SPSS Statistics for Windows, Version 21.0 (IBM Corp., Armonk, New York, United States). Descriptive statistics were expressed as mean±standard deviation and percentage distributions. Categorical variables were compared using cross-tabulation analyses.

Univariate analyses were conducted to compare demographic, clinical, laboratory, and radiological variables between patients with shunt infection and those with mechanical shunt dysfunction. Although multivariable logistic regression analysis was initially planned, a stable model could not be constructed due to the near-complete separation of key inflammatory variables, particularly C-reactive protein (CRP) and white blood cell (WBC) levels.

## Results

Patient distribution and demographic characteristics

During the study period, the medical records of 1,206 patients who underwent ventriculoperitoneal shunt surgery were reviewed. Of these, 390 patients who required surgical revision due to shunt infection or mechanical shunt dysfunction were included in the analysis. Among the included patients, 140 (35.9%) were classified in the shunt infection group, while 250 (64.1%) were assigned to the mechanical shunt dysfunction group.

Male sex was significantly more frequent in the shunt infection group, whereas female sex predominated in the mechanical shunt dysfunction group (χ², p<0.01). Patients younger than one year were more commonly observed in the infection group compared with the mechanical shunt dysfunction group (χ², p<0.05). With respect to hydrocephalus etiology, congenital causes were predominant in both groups; however, this proportion was significantly higher in the mechanical shunt dysfunction group (χ², p<0.01). The demographic and clinical characteristics of the study population are summarized in Table 1.

**Table 1:** Demographic and clinical characteristics of the study population Categorical variables were compared using Pearson's chi-squared test. Values are presented as n (%). The chi-squared (χ²) statistic and corresponding p-values are shown.

Variable	Shunt infection (n=140), n (%)	Mechanical shunt dysfunction (n=250), n (%)	χ²	P-value
Male sex	85 (60.7)	104 (41.6)	13.1	<0.01
Female sex	55 (39.3)	146 (58.4)	-	-
Age <1 year	72 (51.4)	68 (27.2)	18.2	<0.05
Congenital etiology	73 (52.1)	189 (75.6)	21.6	<0.01
Revision within the first 6 months	102 (72.9)	105 (42)	34.8	<0.001

Timing of revision and clinical findings

In the shunt infection group, the majority of surgical revisions were performed within the first six months (102/140, 72.9%). In contrast, revisions in the mechanical shunt dysfunction group occurred significantly later (χ², p<0.001).

With respect to clinical presentation, the presence of fever was significantly more frequent in the shunt infection group compared with the mechanical shunt dysfunction group (99/140, 70.7% vs. 41/250, 16.4%; χ², p<0.001). Clinical findings are summarized in Table 2.

**Table 2:** Clinical, laboratory, and CSF findings Values are presented as n (%). Categorical variables were compared using Pearson's chi-squared test. CSF: cerebrospinal fluid; WBC: white blood cell count; CRP: C-reactive protein

Parameter	Shunt infection (n=140), n (%)	Mechanical shunt dysfunction, n (%)	χ²	P-value
Fever	99 (70.7)	41 (16.4)	78.9	<0.001
Elevated WBC	101 (72.1)	39 (15.6)	96.4	<0.001
Elevated CRP	123 (88.5)	16 (6.4)	165.2	<0.001
Low CSF glucose	72 (51.4)	3 (13)	14.6	<0.001
High CSF protein	107 (76.4)	8 (36)	20.1	<0.001

Laboratory and CSF findings

Laboratory analyses demonstrated that elevated WBC counts and elevated CRP levels were significantly more frequent in the shunt infection group compared with the mechanical shunt dysfunction group (χ², p<0.001 for both). Evaluation of CSF biochemistry revealed that decreased CSF glucose levels (72/140, 51.4%) and increased CSF protein levels (107/140, 76.4%) were significantly more common in the infection group than in the mechanical shunt dysfunction group (χ², p<0.001 for both).

Patients in whom CSF samples were obtained because of initial clinical suspicion but were subsequently considered unlikely to have infection based on clinical assessment and intraoperative findings were classified in the mechanical shunt dysfunction group. In these patients, laboratory and CSF parameters were largely within normal limits. Detailed laboratory and CSF findings are presented in Table 2.

WBC and CRP values of all 390 patients who underwent surgery for shunt malfunction, 140 with shunt infection and 250 with mechanical shunt dysfunction, were summarized in a separate table (Table 3). Pearson's chi-squared analysis demonstrated a statistically significant association between the presence of infection and elevated WBC and CRP levels at the 99.9% confidence level (χ², p<0.001 for both). Accordingly, infection was present in 77.7% of patients with elevated WBC levels, whereas 22.3% had no infection. Similarly, infection was observed in 82% of patients with elevated CRP levels, while 18% had no infection.

**Table 3:** Laboratory findings in patients with shunt infection According to Pearson's chi-squared analysis, a statistically significant association was observed between the presence of infection and elevated WBC and CRP levels at the 99.9% confidence level (χ², p<0.001). Among patients with elevated WBC levels, 77.7% had shunt infection, whereas 22.3% did not. Similarly, shunt infection was present in 82% of patients with elevated CRP levels, while 18% showed no evidence of infection. WBC: white blood cell count; CRP: C-reactive protein

Variable	Level	Infection (+), n	Infection (–), n	OR (95% CI)	P-value
WBC	Normal	39	221	Reference	-
	Elevated	101	29	19.7 (11.1-35.0)	<0.001
CRP	Normal	16	223	Reference	-
	Elevated	124	27	64.1 (33.0-124.5)	<0.001

CSF direct microscopy and culture results

In the shunt infection group, CSF culture positivity was detected in 120/140 patients (85.7%). Among the isolated pathogens, coagulase-negative staphylococci, particularly *Staphylococcus epidermidis*, were identified as the most frequently isolated microorganisms. In contrast, no clinically significant microbial growth was observed in CSF cultures obtained from patients in the mechanical shunt dysfunction group.

On direct microscopic examination of CSF, microorganisms were detected in 111/140 patients (79.3%) with shunt infection, whereas no microorganisms were observed in the remaining 29/140 patients (20.7%). Detailed CSF direct microscopy and culture results are presented in Table 4.

**Table 4:** CSF culture results and distribution of causative pathogens in patients with shunt infection (n=140) Percentages were calculated based on the total number of patients diagnosed with shunt infection (n=140). *Staphylococcus epidermidis *was the most frequently isolated pathogen among coagulase-negative staphylococci. Pearson's chi-squared test was used for categorical comparisons. CSF: cerebrospinal fluid

Parameter	n (%)
CSF culture result	
Positive	120 (85.7)
Negative	20 (14.3)
Microorganism distribution	
Coagulase-negative staphylococci (total)	77 (55)
Staphylococcus epidermidis	60 (42.8)
CSF direct microscopy findings	
Positive	111 (79.3)
Negative	29 (20.7)

Shunt and valve characteristics

Evaluation of shunt and valve characteristics demonstrated that programmable valves were used more frequently in the mechanical shunt dysfunction group compared with medium-pressure valves (70/220, 31.8% vs. 180/986, 18.2%; χ², p<0.001). Although this difference was statistically significant, it was not sufficient to establish a causal relationship. Shunt and valve characteristics are summarized in Table 5.

**Table 5:** Distribution of valve types in patients undergoing ventriculoperitoneal shunt placement Complication rates for programmable and medium-pressure valves were evaluated within each valve type separately. In the univariate analysis, a statistically significant association was identified between valve type and shunt dysfunction (χ²=20.6; p<0.001). The rate of shunt dysfunction was significantly higher in patients with programmable valves (31.8%) compared with those with medium-pressure valves (18.2%) (Pearson's chi-squared analysis).

Valve type	Dysfunction (+), n (%)	Dysfunction (–), n (%)	Total, n	χ²	P-value
Programmable	70 (31.8)	150 (68.2)	220	20.6	<0.001
Medium-pressure	180 (18.2)	806 (81.8)	986	-	-
Total	250	956	1,206	-	-

Abdominal ultrasonography and papilledema findings in shunt dysfunction

Among patients who underwent surgery for mechanical shunt dysfunction, abdominal ultrasonography data were available for 159 cases. Of these patients, no free intraperitoneal fluid was detected in 104/159 cases (65.4%), whereas free fluid was observed in 53/159 cases (33.3%). An abdominal abscess was reported in 2/159 cases (1.3%).

Ophthalmological data regarding papilledema were available for 82 patients. Papilledema was absent in 76/82 patients (92.7%), while evidence of papilledema was observed in 6/82 patients (7.3%). Abdominal ultrasonography and papilledema findings in patients with shunt dysfunction are summarized in Table 6.

**Table 6:** Abdominal ultrasonography findings in patients with mechanical shunt dysfunction Values are presented as n (%). Papilledema was assessed by ophthalmological examination, and abdominal findings were evaluated using ultrasonography. Due to clinical indications, the total number of patients differed between assessments.

Findings	n (%)
Papilledema (n=82)	
Absent	76 (92.7)
Present	6 (7.3)
Abdominal ultrasonography findings (n=159)	
No free intraperitoneal fluid	104 (65.4)
Free intraperitoneal fluid	53 (33.3)
Abdominal abscess	2 (1.3)

Abdominal ultrasonography and papilledema findings in shunt infection

Abdominal ultrasonography and papilledema findings were evaluated in patients who underwent surgery for shunt infection. Abdominal ultrasonography data were unavailable for 42 patients. Among the remaining patients (n=98), free intraperitoneal fluid was detected in 37/98 patients (37.8%), whereas no free fluid was observed in 36/98 patients (36.7%). An abdominal abscess was identified in 25/98 patients (25.5%).

Papilledema status was unavailable for 94 patients. Among those with available ophthalmological data (n=46), papilledema was absent in 43/46 patients (93.5%) and present in 3/46 patients (6.5%). Abdominal ultrasonography and papilledema findings in the shunt infection group are summarized in Table 7.

**Table 7:** Abdominal ultrasonography and papilledema findings in patients with shunt infection Values are presented as n (%). Abdominal findings were evaluated by ultrasonography, and papilledema was assessed by ophthalmological examination. Patient numbers differ between assessments due to unavailable data.

Findings	n (%)
Abdominal ultrasonography (n=98)	
No free intraperitoneal fluid	36 (36.7)
Free intraperitoneal fluid	37 (37.8)
Abdominal abscess	25 (25.5)
Papilledema (n=46)	
Absent	43 (93.5)
Present	3 (6.5)

Shunt order at the time of infection

Among patients who underwent surgery due to shunt infection, the infected shunt was the first shunt in 35.7% of cases, the second shunt in 26.4%, the third shunt in 17.1%, and the fourth or subsequent shunt in 20.8% of patients. The mean number of shunts per patient was 2.38±1.56, with a minimum of 1 and a maximum of 11 shunts implanted in individual patients. These findings are summarized in Table 8.

**Table 8:** Order of the shunt at the time of infection in patients with shunt infection Values are presented as n (%). Percentages were calculated based on the total number of patients with shunt infection (n=140).

Shunt order	n (%)
1st shunt	50 (35.7)
2nd shunt	37 (26.4)
3rd shunt	24 (17.1)
≥4th shunt	29 (20.8)

## Discussion

In the present study, shunt-related complications were evaluated in 1,206 patients who underwent ventriculoperitoneal shunt surgery. Surgical intervention was required due to shunt infection in 140 patients (11.6%) and due to shunt dysfunction in 250 patients (20.7%), yielding an overall shunt failure rate of 32.3%. Reported shunt infection rates in the literature range from 10% to 22% per patient and approximately 6% per procedure; therefore, the infection rate observed in our study is consistent with previously published data and falls within acceptable limits [19-22]. Similar infection rates reported in series with a high proportion of pediatric patients further support our findings [21].

Rates of shunt dysfunction and revision are known to exceed infection rates and to vary widely across studies. Long-term follow-up investigations, particularly in pediatric populations, have reported revision-requiring shunt dysfunction rates of up to 40-50% [9,11]. The shunt dysfunction rate of 20.7% and the overall failure rate of 32.3% observed in our cohort are comparable to the approximately 30-35% overall shunt failure rates reported in the literature [9,10]. Higher rates reported in studies with longer follow-up periods are likely attributable to differences in follow-up duration, definitions of shunt failure, and patient populations [9-11]. In this context, our findings derived from a large cohort with a mixed age distribution reflect real-world clinical practice and align with existing data.

This study comparatively analyzed the demographic, clinical, laboratory, and radiological characteristics of shunt infection and shunt dysfunction in patients undergoing ventriculoperitoneal shunt placement for hydrocephalus. The results demonstrate that these two complications differ substantially in terms of affected patient profiles and clinical and laboratory manifestations at presentation. Unlike many previous reports focusing on a single complication type, our study systematically compares shunt infection and dysfunction within the same cohort, providing clinically relevant insights.

Infection-related findings and comparison with the literature

A predominance of male patients was observed in the shunt infection group. Although prior studies have identified age, prematurity, comorbid conditions, and surgery-related factors as major risk factors for shunt infection, often reporting a balanced or slightly male-predominant sex distribution [23,24], the more pronounced male predominance in our cohort may reflect referral patterns and etiological characteristics of our patient population. In contrast, the predominance of female patients in the shunt dysfunction group suggests that these complications may cluster in distinct demographic subpopulations.

Previous shunt surgery is a well-established risk factor for shunt infection [15,25]. Consistent with this, 64.3% of patients undergoing surgery for shunt infection in our study had a history of multiple shunt procedures, supporting existing evidence that repeated shunt surgeries increase the risk of subsequent infection.

With respect to causative microorganisms, our findings are concordant with prior reports identifying coagulase-negative staphylococci, particularly *Staphylococcus epidermidis*, as the most frequently isolated pathogens in ventriculoperitoneal shunt infections [19,23,26,27]. High rates of CSF direct microscopy and culture positivity in our infection group, with *Staphylococcus epidermidis* identified in 42.8% of cases, further support the concept that shunt infections are predominantly associated with skin flora-derived nosocomial pathogens and underscore the importance of meticulous surgical technique, perioperative antibiotic prophylaxis, and infection control measures [15,16,20-22,26,28-31].

The predominance of infections in the early postoperative period suggests contamination of shunt material during implantation as a major mechanism [20,21,25,26], whereas late-onset infections are more commonly attributed to hematogenous spread or delayed colonization [32,33]. The timing observed in our study aligns with these reports and highlights the importance of close clinical and laboratory surveillance, particularly during the early postoperative phase.

Shunt dysfunction, mechanical complications, and valve type

The predominance of congenital etiology and the high proportion of pediatric and adolescent patients in the shunt dysfunction group are consistent with an increased risk of mechanical failure in patients requiring long-term shunt dependency [7-9,11,24,34-37]. Prior pediatric series have shown that a substantial proportion of revisions are due to mechanical obstruction, distal catheter problems, and valve malfunction [8-11,24,34-37]. In our cohort, shunt dysfunction without evidence of infection was primarily characterized by reservoir (dome) dysfunction and clinical and radiological features consistent with mechanical obstruction, supporting these observations.

The association between valve type and shunt dysfunction remains controversial. While some studies report no significant impact of valve type on shunt longevity, others describe distinct complication profiles for specific valve designs. In our cohort, a higher dysfunction rate was observed with programmable valves compared with medium-pressure valves. This may reflect the greater mechanical complexity of programmable systems and their preferential use in more challenging clinical scenarios. However, confirmation of an independent association would require multivariable analyses. Accordingly, valve selection, particularly in pediatric and congenital hydrocephalus, should consider potential long-term dysfunction risk in addition to theoretical advantages [35,36,38,39]. Given the retrospective design and heterogeneity in clinical decision-making, causal inferences should be made cautiously.

Inflammatory markers, CSF parameters, and differential diagnosis

A key finding of this study is the marked separation between infection and dysfunction with respect to CRP and WBC levels. Elevated CRP and WBC predominated in the infection group, whereas these markers were largely normal or only mildly elevated in the dysfunction group. When combined with decreased CSF glucose and increased CSF protein levels, these findings constitute a strong biochemical profile favoring infection. Although the diagnostic value of CSF biochemistry and cell counts is well established [40-42], atypical patterns and culture-negative infections, particularly in pediatric patients, have been reported [10,28,43]. In our study, high culture positivity and strong concordance between CSF and systemic inflammatory markers suggest that combined interpretation of these parameters provides a reliable approach to differential diagnosis.

In contrast, the absence of infection-compatible CSF findings in the dysfunction group supports the concept that mechanical dysfunction is a process independent of infection. While CSF analysis is valuable in distinguishing infection from dysfunction, indiscriminate invasive procedures carry inherent risks [44-47]. Our findings indicate that, when clinical and radiological features do not favor infection, integrating laboratory results with mechanical assessment (e.g., reservoir function and imaging) may reduce unnecessary invasive interventions.

Radiological findings: ultrasonography, abdominal complications, and papilledema

Abdominal complications and pseudocyst formation are recognized ventriculoperitoneal shunt-related problems with variable incidence [48-53]. In our study, abdominal abscesses were more frequent in the infection group, whereas true abscess formation was rare in the dysfunction group (1.3%). Notably, several dysfunction cases had ultrasonographic findings suggestive of abscess without intraoperative or clinical confirmation, underscoring the need to interpret abdominal ultrasonography in conjunction with clinical and laboratory data and to consider false-positive imaging results. Accordingly, abdominal ultrasonography should be regarded as a supportive, not definitive, diagnostic modality in shunt evaluation.

Despite its utility in identifying distal catheter migration, pseudocyst formation, peritonitis, and abscess, the occurrence of dysfunction in patients with free intraperitoneal fluid suggests mechanisms such as impaired peritoneal absorption or hyperacute dysfunction. The limited specificity of imaging and challenges in differentiating pseudocyst from abscess have been noted previously [48-50,52].

Papilledema, while a classic sign of elevated intracranial pressure, has limited sensitivity, particularly in pediatric hydrocephalus and chronic conditions [54-56]. The low prevalence of papilledema in both groups in our study aligns with reports indicating its absence in infants with open fontanelles, slowly progressive pressure elevation, or preserved clinical compensation [54-56]. Thus, papilledema alone is insufficient for differentiating infection from dysfunction or for reliably reflecting intracranial pressure severity and should be interpreted alongside other clinical and radiological findings.

The principal strengths of this study include the use of a large single-center cohort managed by the same surgical team with similar follow-up protocols, the systematic comparison of shunt infection and shunt dysfunction within the same dataset, and the comprehensive evaluation of demographic, laboratory, CSF, radiological, and valve-related parameters. However, several limitations should be acknowledged. These include the retrospective design of the study, incomplete data availability for certain variables, and limited control over clinical factors influencing valve selection, such as surgical indications and associated comorbidities. In addition, as a single-center study, the generalizability of the findings to other institutions and patient populations should be interpreted with caution. This study has several limitations. First, its retrospective design may introduce selection and information bias. To minimize these effects, predefined inclusion and exclusion criteria were applied, objective laboratory and CSF parameters were used, and standardized definitions for shunt infection and mechanical shunt dysfunction were adopted. In addition, the single-center design ensured consistency in diagnostic evaluation and surgical decision-making. Future prospective multicenter studies with standardized diagnostic criteria and follow-up protocols are warranted to reduce bias further and improve the generalizability of the findings.

## Conclusions

The findings of our study highlight the critical role of WBC count, CRP levels, CSF biochemistry, and culture results in addressing the frequently encountered clinical question of whether a patient presents with shunt infection or shunt dysfunction. In cases in which signs of infection are not prominent and inflammatory markers remain within normal limits, prioritizing mechanical evaluation and planning targeted revision, rather than initiating unnecessary antibiotic therapy or complete shunt removal, may represent a more rational and patient-centered approach. Conversely, in patients with concomitantly elevated CRP/WBC levels, abnormal CSF biochemistry, and positive culture results, rapid recognition of infection and implementation of aggressive treatment strategies may reduce morbidity.

In conclusion, our study demonstrates that shunt infection and shunt dysfunction should be considered not merely as complication categories but as distinct clinical phenotypes, necessitating a multiparametric approach to differential diagnosis. Validation of this approach through future prospective and multicenter studies may further optimize valve selection, infection prophylaxis, and revision strategies in patients undergoing ventriculoperitoneal shunt surgery.
